# Resident Physicians Have an Increased Risk of Adverse Driving Events Following Extended-Duration Work Shifts: A Systematic Review

**DOI:** 10.7759/cureus.73922

**Published:** 2024-11-18

**Authors:** Nicola Bonnell, Kaiz Jamal, Bianca Boicu, Jeffrey R Brubacher

**Affiliations:** 1 Department of Emergency Medicine, University of British Columbia, Vancouver, CAN

**Keywords:** car driving, driving ability, motor vehicle crash (mvc), residency and internship, work hours

## Abstract

Resident physicians often work extended-duration work shifts (EDWSs) exceeding 16 hours. EDWSs are associated with fatigue, workplace errors, mental health problems, and motor vehicle incidents. A 2019 systematic review reported that resident physicians had an increased risk of motor vehicle collisions (MVCs) and of falling asleep at the wheel after EDWSs. This systematic review updates those findings with recent literature.

Embase, PubMed, Cochrane Database, and Ovid Medline were searched for original research articles studying resident physician driving safety following EDWS. Two authors independently reviewed articles for inclusion. Both reviewers performed data extraction and quality appraisal for each included article.

Six articles met the inclusion criteria. Three articles found associations between EDWS and increased sleepiness in resident physicians. Self-reported sleepiness was increased by 46% following an EDWS compared to a normal-length shift. Objective measures of sleepiness were also increased following an EDWS. Similarly, there was a three-fold increase in adverse driving events following an EDWS compared to pre-EDWS. One study found 3.90 higher odds of an adverse driving incident following an EDWS compared to a day shift.

EDWS are associated with an increased risk of adverse driving incidents, including collisions and falling asleep while driving, in resident physicians. Possible solutions including compensation for ride-share and taxi services, scheduled breaks, education on risks of driving while fatigued, and the use of caffeine may lower the risk of adverse driving incidents post-EDWSs. Further research is needed to assess the impact of possible solutions.

## Introduction and background

Prolonged medical resident work hours result in numerous safety concerns, including increased workplace errors leading to detriments in patient safety [1,2]. Prolonged work hours also impact medical residents themselves by causing issues such as fatigue, mental health problems, dangerous driving, and motor vehicle incidents [1,2].

While the United States has implemented limitations for resident work hours, Canadian guidelines are set on a provincial basis, based either on hours per week or continuous hours spent in hospital [3,4]. In 2003, the American Accreditation Council for Graduate Medical Education (ACGME) implemented an 80-hour workweek limitation for U.S. medical residents [3]. In 2011, they restricted first-year resident shift duration to a maximum of 16 consecutive hours [3]. This was done in an effort to improve resident well-being and patient safety, as well as to decrease physical and mental burnout [3-5]. Despite these measures, resident work shifts in the U.S. often breach these limitations, regardless of specialty [6,7]. Residents may underreport duty hours to adhere to the ACGME limits [8,9]. As such, medical residents continue to be placed in situations where fatigue is prominent, which can have a negative impact on their safety and well-being. Numerous studies continue to report high levels of sleep deprivation, fatigue, and burnout in medical residents, especially when they work extended-duration work shifts (EDWSs), defined as shifts longer than 16 hours [3,10-13].

Sleep loss is correlated with driving impairment and unsafe motor vehicle operation [14-17]. Night shift workers have increased drowsiness, near-crashes, and lane excursions after a night shift compared to after a night of normal sleep [18]. During the commute home after a night shift, participants reported more sleepiness and had increased rates of eye closures and motor vehicle accidents compared to after a normal night of sleep [19]. Nurses reported a significant increase in sleepiness and driving events on the post-night shift commute as compared to the pre-shift commute [20]. The consistency in these findings provides a rationale for studying the impact of EDWSs on driving incidents in medical residents.

A 2019 systematic review found increased rates of motor vehicle collisions (MVCs) and self-reported incidents of falling asleep at the wheel in medical residents after EDWSs [21]. Since then, several new studies have investigated the impact of extended work shifts on driving safety in medical residents. This manuscript updates the previous review and summarizes recent literature surrounding EDWSs and driving safety in medical residents.

## Review

Methods

This review was registered with PROSPERO (CRD42023366066) and PRISMA guidelines were followed. For consistency with the previous systematic review by Mak et al. (2019), the same search terms and databases were used to identify articles for this review [21]. This review includes new articles published since Mak’s review (i.e., February 2018 to May 2024). The searches were conducted in Embase, PubMed, Cochrane Database, and Ovid Medline. The search combined terms related to (“Driving”) AND (“Resident*” OR “Intern*”) AND (“Work hour*” OR “Workload” OR “On call” OR “Shift work” OR “Sleep deprivation”). Please refer to Mak et al. for additional search details [21].

Included manuscripts must have studied the impact of EDWSs (defined as shifts longer than 16 hours) on the driving safety of resident physicians in any specialty and any year of postgraduate training. Specifically, we included manuscripts with outcomes that included traffic accidents or dangerous driving (such as falling asleep at the wheel). The manuscripts must have compared driving outcomes following an EDWS versus after shifts of normal duration (8 hours) or studied the association of driving outcomes with the number of EDWSs worked in a certain time period. Articles were excluded if they did not report original research, if the duration of work shifts was under 16 hours or undefined, or if published in a language other than English or French.

Covidence® was used to assist with data collection and study organization [22]. Two reviewers reviewed the titles and abstracts for 200 articles and inter-rater agreement was measured. Each reviewer then independently screened approximately half of the articles by title and abstract and excluded those that did not meet the above criteria. The remaining articles underwent a full-text review by two reviewers to determine eligibility; inter-rater agreement for the full-text review was measured. All selected manuscripts were assessed using the Newcastle-Ottawa Scale (NOS) to determine the risk of bias with respect to selection, comparability, and exposure [23]. All selected manuscripts were read in depth by two reviewers and relevant data was extracted using a predetermined datasheet. Extracted data included: (i) title, (ii) author(s), (iii) year of publication, (iv) study participants, (v) control and comparison groups, (vi) adverse driving incidents, and (vii) risk of adverse driving outcomes associated with EDWSs. Individual studies were summarized, but a quantitative synthesis was not possible due to heterogeneous methods and outcomes between studies.

Results

Search Results and Characteristics of Included Studies

**Figure 1:**
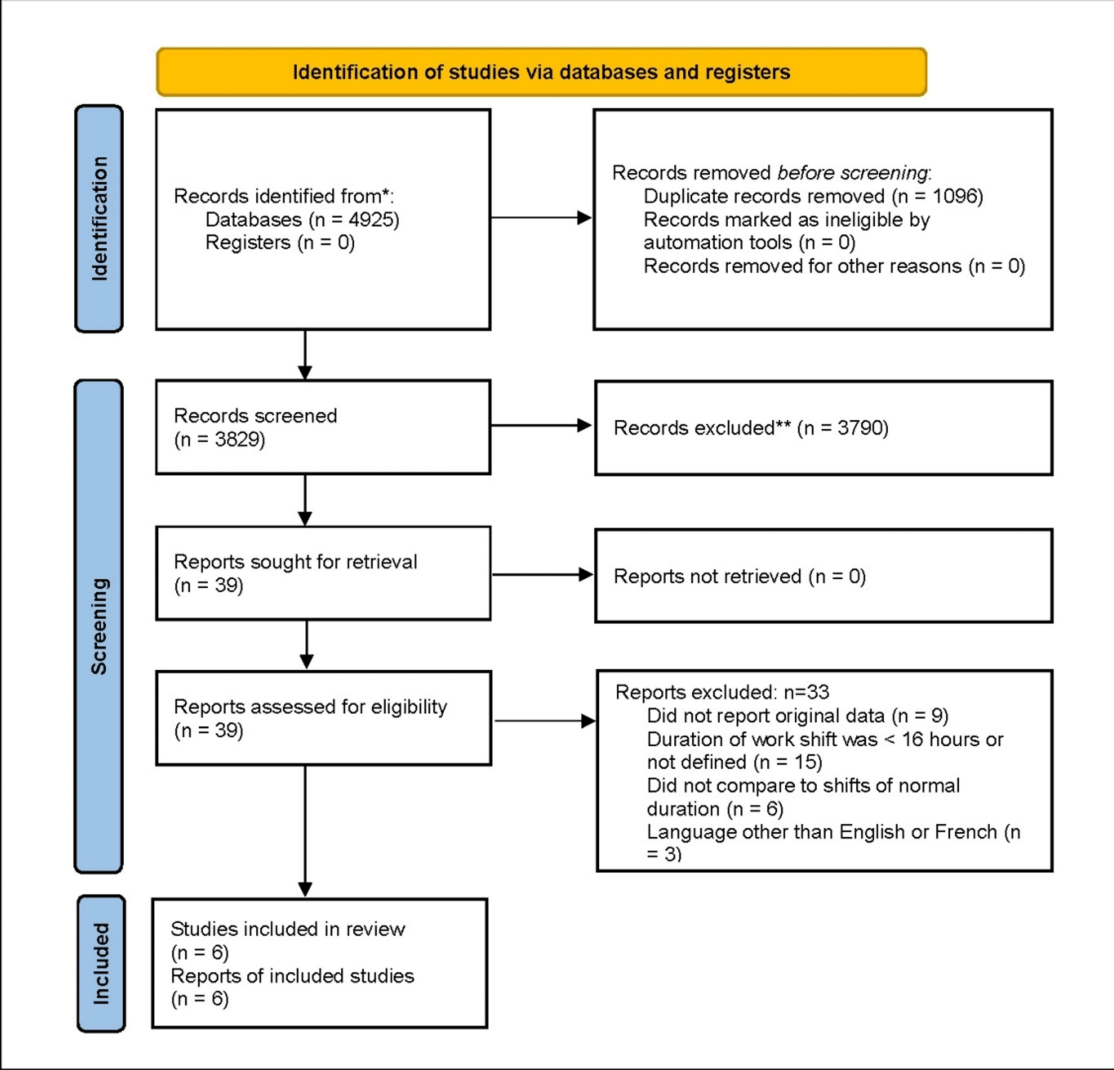
PRISMA diagram for the article screening and selection process PRISMA: Preferred Reporting Items for Systematic Reviews and Meta-Analyses

After removing duplicates, a total of 3829 studies were found. Of these, 3823 were excluded and 6 were included in the final review. Figure 1 shows the Preferred Reporting Items for Systematic Reviews and Meta-Analyses (PRISMA) flow diagram. Inter-rater reliability was substantial for both abstract and title screening (Kappa = 0.86) and full-text review (Kappa = 1.0).

Three of the six included articles were prospective, the rest were retrospective. Study characteristics and findings are summarized in Table 1. To summarize all available literature in one place, the table includes the five manuscripts from the 2019 review, together with the six manuscripts from the current review. Of the six included studies from the current review, one was from Canada, four from the USA, and one from Peru. Post-graduate trainees from both surgical and medical specialties were included. Anderson et al. (2018) included 16 residents from the internal medicine, surgery, anesthesia, and pediatric specialties [24]. Cassidy et al. (2021) included 273 trainees from all specialties [25]. Freedman-Weiss et al. (2020) and Schlick et al. (2021) included 58 and 7391 general surgery residents, respectively [26,27]. Weaver et al. (2020) included 15, 276 first-year residents but did not define their specialties [28]. Lastly, Villafuerte-Trisolini et al. (2017) included 10 interns but did not list their specialties [29]. The included studies evaluated driving outcomes including self-reported near-misses, MVCs, or falling asleep while driving as well as objective measures like reaction times. The risk was determined by comparing outcomes after EDWSs to outcomes after shifts lasting less than 16 hours or to pre-shift outcomes.

**Table 1:** Characteristics and findings of included studies (from Mak et al. and the current review)

Author (Year)	Study Location	Study Type	Population	Risk of Bias (Newcastle-Ottawa Scale (NOS))	Sleepiness	Driving Outcomes
Barger et al. (2005) [30]	USA	Prospective repeated-measures survey.	2737 first-year residents.	5	OR falling asleep at the wheel if working > 5 EDWS vs. 0 EDWS = 2.39 (95% CI, 2.31 to 2.46).	OR for MVC after EDWS versus after regular hours shift = 2.3 (95% CI, 1.6 to 3.3). OR for near-miss after EDWS versus after regular hours shift = 5.9 (95% CI, 5.4 to 6.3). Increase in risk for MVC on commute home per EDWS worked per month = 16.2% (95% CI, 7.8 to 24.7%).
Marcus et al. (1996) [31]	USA	Retrospective survey.	61 pediatric residents and 74 attending physicians.	3	44% of residents reported falling asleep at the wheel at traffic lights vs. 12.5% of attending physicians on their commute home post-EDWS (p<.001)	40% of reported MVCs occur when residents are post-call.
O’Grady et al. (2012) [32]	Australia and New Zealand	Retrospective survey.	659 surgery residents.	3	Residents who reported never falling asleep at the wheel post-EDWS lost fewer total sleep hours than residents who did report falling asleep at the wheel. Momentary dozing while driving is more likely in residents with >5.5 h of weekly sleep loss hours (p<.05)	Not reported.
Ware et al. (2006) [33]	USA	Experimental study, within-subjects design.	22 medicine residents and 1 student.	5	Not reported.	Significant increase in MVC frequency after overnight calls in male residents only.
Tornero et al. (2012) [34]	Spain	Experimental study, within-subjects design.	25 emergency medicine residents.	5	Not reported.	No significant difference in standardized Driver Test results (ASDE test) after EDWS compared to test results after 7 hours of rest.
Anderson et al. (2018) [24]	Boston, USA	Prospective.	12 internal medicine residents, 2 surgery residents, 1 anesthesiology resident, and 1 pediatrics resident.	7	Self-reported sleepiness elevated by 46% on post-EDWS commute compared to a 1.3% elevation post-normal duration shift commute (p<.0001)	Increased number of self-reported adverse driving events on post-EDWS commute compared to post-normal duration shift commute (1.1 ± 0.78 versus 0.59 ± 0.63, p<.005)
Cassidy et al. (2021) [25]	British Columbia, Canada	Retrospective.	216 residents in a medical discipline and 57 residents in a surgical discipline.	7	Not reported.	Increased likelihood of self-reporting an MVC for every additional ten hours of work per week (OR=1.69, 95% CI: 1.06-2.77, p=.0306).
Freedman-Weiss et al. (2021) [26]	Connecticut, USA	Retrospective.	58 general surgery residents at Yale University School of Medicine.	7	75.9% of residents experienced their greatest fatigue after a 24-hour shift compared to 20.7% experiencing their greatest fatigue driving to work.	Not reported.
Schlick et al. (2021) [27]	USA	Retrospective.	7391 general surgery residents.	7	Not reported.	Residents working shifts greater than 28 hours ≥ 3 times in the last month were more likely to report falling asleep while driving (53.1% versus 31.8%, p<.001) and to report near-miss mvcs versus p compared residents who worked the same duration shift times in last month.
Weaver et al. (2020) [28]	USA	Prospective.	15276 first-year residents across any specialty.	7	Not reported.	Compared to working no EDWS, working more than five EDWS per month was associated with an increased risk of MVC (RR 2.02, p=.03), near-crashes (RR 1.45, p=.0022), and attentional failures (RR 1.71, p<.0001)
Villafuerte-Trisolini et al. (2017) [29]	Peru	Prospective.	10 resident physicians across any medical specialty.	8	Self-reported sleepiness scores using the Daily Fatigue Impact Scale were worse 1-day post-EDWS compared to during the EDWS (16.6 ± 7.3 versus 7.8 ± 3.0, p=.005).	Post-EDWS, drivers who did not report adverse driving outcomes were found to have faster reaction times (335.87 versus 444.27) and no lapses in reaction time test (0 versus 5) compared to those who did report adverse driving outcomes.

Reported Measures of Sleepiness

Three included articles used self-reported measures of sleepiness to determine the impact of EDWS on medical residents’ driving behaviors [24,26,29]. Anderson et al. (2018) used the Karolinska Sleepiness Scale to determine self-reported sleepiness and found that 75% of participants reported six or more signs of sleepiness on the drive home from work, compared to only 12% on the drive to work [24]. Self-reported sleepiness was increased by 46% on the commute home after an EDWS compared to 1.26% after a day shift (p < .0001) [24]. Objectively, the Johns Drowsiness Scores (measures involuntary eye blinks and long eye closures) increased by 48% following an EDWS compared to a 25% decrease after a day shift (p = .003) [24]. Freedman-Weiss et al. (2020) found that 75.9% of 58 residents experienced their greatest fatigue after a scheduled 24-hour shift as compared to 20.7% of residents who experienced their greatest fatigue driving to work [26]. Lastly, Villafuerte-Trisolini et al. (2017) determined that scores on the Daily Fatigue Impact Scale were higher the day following an EDWS compared to during the EDWS (16.6 ± 7.3 versus 7.8 ± 3.0, p = .005) [29]. Interns had progressive fatigue during the EDWS and were sleepier while driving or stuck in traffic on their commute home.

Driving Outcomes

All six included articles analyzed the difference in adverse driving events following an EDWS compared to a regular-length shift or the pre-EDWS commute [24-29]. Anderson et al. (2018) reported a three-fold increase in the average number of self-reported adverse driving events (e.g. falling asleep while driving, being distracted, swerving violently, driving through a stop light) when driving home from an EDWS compared with driving to work (1.1 ± 0.78 versus 0.37 ± 0.49 events/drive, p < .005, respectively). Adverse driving events after an EDWS were also significantly increased compared to driving to or from a regular day shift (0.35 ± 0.53 and 0.59 ± 0.63 events/drive, respectively) [24]. The odds of reporting an adverse driving event following an EDWS compared to following a day shift was 3.90 (p = .001) [24]. Cassidy et al. (2021) found that trainees with longer commutes were more likely to report a motor vehicle incident (i.e. falling asleep while driving, sudden braking or swerving, running a red light or stop sign, or a motor vehicle collision) (OR = 1.54, p = .0058). They were also more likely to report an MVC for every additional 10 hours per week of being on-call (OR = 1.69, p = .03) [25]. Schlick et al. (2021) found that general surgery residents who reported working shifts longer than 28 hours three or more times in the last month were more likely to report “nodding off” while driving (53.1% versus 31.8%, p < .001) and near-miss MVCs (44.1% versus 23.7%, p < .001) as compared to residents who worked > 28-hour shifts less than three times a month [27]. Weaver et al. (2020) found that the risk of MVC decreased by 24% after the implementation of the 16-hour ACGME limit, near-crashes decreased by 44%, and attentional failures decreased by 18% [28]. Working an EDWS was independently associated with an increased risk of MVC (likelihood ratio 2.02, p = .03), near-crashes (likelihood ratio 1.45, p = .0022), and attentional failures (likelihood ratio 1.71, p < .0001) [28]. Following an EDWS, Villafuerte-Trisolini et al. (2017) found that drivers who did not report falling asleep in traffic had faster reaction times (335.87 ms versus 444.27 ms; p = .01) and no lapses (0 versus 5) compared to those who did fall asleep [29]. Reaction times increased (worsened) during the second half of the EDWS (p = .01) [29].

Quality Assessment

Details on the quality analysis conducted using the NOS are summarized in Table 1. All six newer studies scored at least seven out of nine stars using the NOS. All studies used self-reporting for measures of outcome and may have been susceptible to recall and reporting bias. However, Villafuerte-Trisolini et al. (2017) also used objective measures of reaction times thus gaining them an extra star on the NOS.

Discussion

This literature demonstrates that, regardless of the outcomes measured or the specialty of the physician-in-training, EDWSs are associated with increased reports of sleepiness and more adverse driving events. These findings are similar to findings from other disciplines in which driving safety and shift work have been studied. Following night shifts, nurses had significantly greater lane deviation compared to day shift participants, and were at increased risk of MVC, even after having three days off work [35]. Police officers who worked 20-hour shifts had decreased reaction times, increased lapses in concentration, and experienced more fatigue compared to those working 10-hour shifts [36].

Despite the ACGME's intent to limit resident work hours to no more than 80 hours per week, it is not uncommon to see this limit being breached [6,7]. The reason adverse driving events increase following EDWSs is likely due to increased sleepiness, which is compounded not only by the duration of the shift but also by the number of shifts worked [24-27]. Junior residents, who were not as accustomed to the longer work hours, were found to have an increased risk of adverse driving events compared to senior residents [26]. Factors such as longer travel time, highway driving, and further travel distance were also correlated with motor vehicle incidents [25,26].

Several researchers have suggested ways to minimize the impact of an EDWS on dangerous driving in medical residents. One proposed solution is for residency programs to provide transportation vouchers or free access to taxis and rideshare services for post-call residents [25,27,28]. Only 6.9% of surgical residents reported using rideshare services when fatigued following a shift; however, 87.7% indicated that they would use rideshare services if reimbursed [26]. Using self-reported measures of fatigue post-EDWS may offer another solution to this problem. By being able to identify their fatigue levels, which correlate with driving outcomes, residents may be able to identify and implement other interventions such as alternative transportation, caffeine, or sleeping at the hospital prior to the commute home [24]. In a study looking at preventing fatigue in shift workers from industries like aircraft maintenance and industrial work, solutions that significantly reduced post-shift fatigue and insomnia included scheduled 20-minute naps, exposure to sunlight and wholefood, nutritious diets, including vegetables, fruits, and low-carb food [37]. Similarly, in nurses working night shifts, Cyr et al. (2023) found that evening bright light exposure prior to their shift resulted in a reduction in sleepiness and work-related errors [38].

This manuscript has several strengths. We conducted a comprehensive review to analyze the impact of EDWSs on adverse driving events on medical residents. Findings were consistent across several medical specialties and from studies conducted in different countries, thus emphasizing the strong association between EDWSs and adverse driving events. The consistency of the findings from the Mak et al. (2019) review with those from the newer studies identified in our review reinforces this conclusion. There are also limitations. Most included studies were from the US, and resident duties and responsibilities may vary between countries. Additionally, a majority of the reports used retrospective self-reports to evaluate outcomes; self-reports are subject to a recall and reporting bias. Further, there were no objective measures of actual sleep hours, nor were confounding variables controlled for, including length of commute, highway versus city driving, vehicle type, driving experience, or analysis of shift responsibilities. That said, the included studies consistently reported that EDWSs increased sleepiness and resulted in an increased risk of adverse driving events compared to non-EDWSs. These findings appear to be universal and warrant concern, thus emphasizing the need for solutions.

Future research needs to be done to design and evaluate interventions to prevent adverse driving events post-EDWS. These may include scheduled breaks during or after an EDWS, varying time periods between EDWSs, or making ride-share or taxi services available to medical residents. Further, improving the quality of previous studies by controlling for confounding variables and designing prospective studies that limit recall bias may help strengthen our understanding of how EDWSs impact driving safety.

## Conclusions

We conducted a systematic review of all articles investigating EDWSs and driving outcomes published from February 2018. This review extends findings from a previous review and shows that EDWSs continue to be associated with elevated levels of sleepiness in residents and an increased risk of falling asleep while driving, MVCs, near-misses, and overall driving impairment. Suggested solutions include free access to ride-share programs, educating residents on the impacts of driving while fatigued, and using self-reported measures of fatigue post-EDWS to determine whether the drive home could be completed safely. Future research should evaluate interventions that aim to ensure that work conditions do not place medical trainees at a higher risk of unsafe driving.
